# FeCoSe_2_ Nanoparticles Embedded in g-C_3_N_4_: A Highly Active and Stable bifunctional electrocatalyst for overall water splitting

**DOI:** 10.1038/s41598-020-63319-7

**Published:** 2020-04-14

**Authors:** Muhammad Zulqarnain, Afzal Shah, Muhammad Abdullah Khan, Faiza Jan Iftikhar, Jan Nisar

**Affiliations:** 1 Department of Chemistry Quaid-i-Azam University, 45320 Islamabad, Pakistan; 2 Renewable Energy Advancement laboratory, Department of Environmental Sciences, Quaid-i-Azam University, 45320 Islamabad, Pakistan; 3 Department of Chemistry, College of Science, University of Bahrain, Sakhir, 32038 Kingdom of Bahrain; 4 NUTECH School of Applied Sciences and Humanities, National University of Technology, Islamabad, 44000 Pakistan; 5 National Centre of Excellence in Physical Chemistry, University of Peshawar, Peshawar, 25120 Pakistan

**Keywords:** Electrochemistry, Energy, Materials chemistry

## Abstract

To investigate cost affordable and robust HER and OER catalysts with significant low overpotentials, we have successfully embedded FeCoSe_2_ spheres on smooth surfaces of graphitic carbon nitride that demonstrated high stability and electrocatalytic activity for H_2_ production. We systematically analyzed the composition and morphology of Fe_x_Co_1−x_Se_2_/g-C_3_N_4_ and attributed the remarkable electrochemical performance of the catalyst to its unique structure. Fe_0.2_Co_0.8_Se_2_/g-C_3_N_4_ showed a superior HER activity, with quite low overpotential value (83 mV at −20 mA cm^−2^ in 0.5 M H_2_SO_4_) and a current density of −3.24, −7.84, −14.80, −30.12 mA cm^−2^ at 0 V (vs RHE) in Dulbecco’s Phosphate-Buffered Saline (DPBS), artificial sea water (ASW), 0.5 M H_2_SO_4_ and 1 M KOH, respectively. To the best of our knowledge, these are the highest reported current densities at this low potential value, showing intrinsic catalytic activity of the synthesized material. Also, the catalyst was found to deliver a high and stable current density of −1000 mA cm^−2^ at an overpotential of just 317 mV. Moreover, the synthesized catalyst delivered a constant current density of −30 mA cm^−2^ for 24 h without any noticeable change in potential at −0.2 V. These attributes confer our synthesized catalyst to be used for renewable fuel production and applications.

## Introduction

Significant increase in the fossil fuel consumption and associated environmental threats of greenhouse gases have urged the scientific community to explore a sustainable and everlasting source of energy^1^. The fuel cell technology is considered as an efficient, long-lasting and environmental friendly source of energy. Among various existing forms of fuel cells, the H_2_/O_2_ fuel cells are deemed as zero carbon discharging technology with only water as the combustion product^2,3^. To ensure higher cell efficiency, the supply of pure fuel is essential and water electrolysis is one of the most convenient ways of producing H_2_. In fact, water splitting has garnered enough spotlight because of the highly pure and plentiful supply of fuel produced during this process. Likewise, this strategy does not demand elevated temperatures and pressure reactors. The overall water electrolysis can be explained by the following two half-cell reactions: hydrogen evolution reaction (HER) at the cathode^4,5^ and oxygen evolution reaction (OER) at the anode^6,7^, investigated in acidic and alkaline conditions, respectively^5,8^.

$${{\rm{2H}}}_{(aq)}^{+}+2{{\rm{e}}}^{-}\to {{\rm{H}}}_{2(g)}$$

$${{\rm{4OH}}}_{(aq)}^{-}\to {{\rm{2H}}}_{2}{{\rm{O}}}_{(l)}+{{\rm{O}}}_{2(g)}{+{\rm{4e}}}^{-}$$

The noble metals i.e., Platinum for HER^9–14^ and Iridium or Ruthenium for OER^15–18^ being expensive and scarce, must be substituted by earth abundant and cost effective non-noble metals^19^ or metal free catalysts such as carbon based electrocatalysts. This work is an effort to substitute the expensive noble metals with only a small amount of earth abundant and cost effective non-noble metal catalysts to achieve better HER activity than the commercial catalysts reported so far. Among the metal free substrates, Carbon Nitride (C_3_N_4_) is the best choice as it offers π-conjugated graphitic planner layers with good conductivity and large surface area^20,21^. The C_3_N_4_ is reported as one of the primitive synthetic polymeric materials that offers different ways to modify its reactivity without any change in composition^22–28^. The g-C_3_N_4_ is believed to possess N-bridged poly(tri-s-triazine) planar structure with high degree of condensation. The s-triazine framework makes it thermally (up to 600 °C) and chemically stable. Moreover, it is a semiconducting material of medium band gap value (2.7 eV) that finds extensive use as a photo(electro)catalyst^29–33^.

Recently, transition-metals sulfides, phosphides and selenides have been found to be efficient HER catalysts^34,35^. Likewise, various electrocatalysts including metal alloys^36,37^, metal nitrides^38,39^, metal phosphides^40^, metal carbides^41^, metal dichalcogenides^42^ and carbon-based catalysts have also attracted attention of researchers in the last few years^43,44^. The transition metal dichalcogenides (TMDs) have been found to be promising water splitting electrocatalysts and have thus gained significant interest in technological applications due to their unique electronic structure, different chemical compositions and diverse crystal symmetry. Several routes i.e. homogeneously dispersed electrocatalyst on conductive supports, chemical vapor and electron beam deposition have been developed to exploit the electrocatalytic properties of these materials^45–47^. However, the above-mentioned synthetic approaches are unfeasible for large scale production as control over the layers, chirality and influence of different substrates on its fabrication is still an area that is not well explored. The chemical doping is considered as an important structure-engineering technique to increase active sites and conductivity of these electrocatalysts^48^ which will be explored in this work.

Previously, Transition metal dichalcogenides (TMDs) such as NiS_2,_ FeS_2,_ and CoS_2_ displayed efficient overall water splitting performance^49–51^. Similarly, Iron (Fe) found in its natural form such as Nitrogenases and Hydrogenase (natural hydrogen evolution enzymatic catalysts) is the most promising material for HER. Hence, Iron doping is expected to increase the electrocatalytic performance of HER catalysts. Similarly, sulfur component of above-mentioned catalysts can be replaced with selenium. Sulfur and selenium are present in the same group and both exhibit similar properties, but selenides are expected to enhance HER performance of electrocatalysts due to low bond strength of Se–H (276 kJ/mol) than P–H (322 kJ/mol) and S–H (363 kJ/mol). Obviously, the bond strength has a great influence on the extent of adsorption/desorption of the intermediate states during catalysis. The weaker Se–H bond as compared to P–H and S–H is expected to facilitate the product desorption from catalytic sites which circumvents catalyst poisoning and thus stable and efficient performance is projected. Moreover, the Se sites can help to promote the delivery of oxygen molecules because of the localized negative charge of Se sites for OER. Likewise, there is a 3d–2p electronic repulsion amid metal and Se d-band centers^32^.

Herein, we have reported a facile method for synthesizing highly active and stable FeCoSe_2_ on graphitic carbon nitride substrate (Fe_x_Co_1−x_Se_2_/g-C_3_N_4_) as novel catalyst for overall water splitting. The catalytic activity of Fe_x_Co_1−x_Se_2_/g-C_3_N_4_ significantly improved with increased Fe percentages in the composite material. The Fe_0.2_Co_0.8_Se_2_/g-C_3_N_4,_ has proven itself to be a promising electrocatalyst for HER and OER due to its high conductivity, unique structure and high surface area.

## Experimental Section

### Chemicals and materials

Iron (III) Nitrate (Fe(NO_3_)_3_·9 H_2_O), Cobalt (II) Chloride hexahydrate (CoCl_2_.6H_2_O), Urea (CO(NH_2_)_2_) and Selenium Powder (Se) were purchased from Sigma-Aldrich. Potassium hydroxide, Sulfuric acid and Iso-propanol were of analytical grade and were used as received without any further purification. Preparation of Indian standard artificial sea water (ASW) and Dulbecco’s Phosphate-Buffered Saline (DPBS) is detailed in Supplementary note 1.

### Synthesis of graphitic carbon nitride

The graphitic carbon nitride was synthesized by using combustion method. The 10 g of urea powder was placed in a covered silica crucible and calcined at 500 °C for 4 h at a heating rate of 2 °C/min. The g-C_3_N_4_ powder was collected and exfoliated in excess of distilled water for 5 h, dried at 80 °C and stored for further use.

### Synthesis of CoSe_2_ and Fe_x_Co_1−x_Se_2_ embedded in g-C_3_N_4_

To synthesize CoSe_2_ embedded in g-C_3_N_4,_ a simple hydrothermal route was followed. Solution A was prepared by adding 0.8 mmol of cobalt source, 2 ml of 0.5 M aqueous solution of ethylene diamine tetra-acetic acid ligand, and 20 mg of g-C_3_N_4_. While solution B was prepared by dissolving 1.62 mmol of Se powder in 8 ml of 3.3 M NaOH. Both solutions were mixed by ultrasonication and the mixture was shifted to a Teflon autoclave and maintained at 180 °C for 18 h. While to produce Fe_x_Co_1−x_Se_2_/g-C_3_N_4_, cobalt source in the solution A was substituted by appropriate amount of iron nitrate and cobalt chloride solutions. In each case, black product was collected and washed with deionized water followed by air drying for characterizations and application of the as-synthesized product. Total quantity of metal sources (Fe + Co) was kept very low and constant (0.8 mmol). For Fe_0.2_Co_0.8_Se_2_/g-C_3_N_4_, the molar ratio of Fe, Co, and Se was maintained at 1: 4: 10. Finally, feeding ratios of metals were further confirmed by average results of EDX (Energy Dispersive X-ray Spectroscopy).

### Fabrication of electrodes

3 mg of each sample and 20 μL of 5 wt.% Nafion were dispersed in 80 μL of iso-propanol to make a slurry. Then 5 μL of slurry was drop casted on glassy carbon electrode (GCE) of geometric surface area of 0.0707 cm^2^ and dried in air. These fabricated electrodes (with mass loading of 2.12 mg cm^−2^) were used as cathodes and anodes for respective electrochemical measurements of HER and OER respectively.

### Characterization

To identify the crystalline structure of products, X-ray diffraction (XRD) was performed on Rigaku D/max 2500 with a Cu Kα1 radiation source (λ = 1.54056 Å). Morphologies of prepared samples were identified at Hitachi S-4800 Scanning Electron Microscope (SEM) at an accelerating voltage of 30 KV. Energy dispersive X-ray spectra (EDX) were observed on an Oxford Material Analysis equipped on SEM and Transmission electron microscopy (TEM). While, Fourier Transform Infra-Red (FTIR) spectroscopic measurements were carried on TENSOR-27 (Bruker) and differential scanning calorimetry/thermal gravimetric analysis (DSC/TGA) on TA SDT Q600.

### Electrochemical measurements

Electrochemical studies were carried out on Autolab PGSTAT302N Metrohm, (running with NOVA 1.11 software) electrochemical workstation at room temperature. While, EIS measurement was made with a Gamry EIS 300 electrochemical impedance analyzer with frequencies ranging from 100 kHz to 0.1 Hz at a voltage amplitude of 10 mV. The electrocatalytic ability of all materials was studied in 0.5 M H_2_SO_4_, DPBS, ASW, and in 1 M KOH for HER using a three-electrode configuration at a scan rate of 20 mV/s. The Fe_x_Co_1−x_Se_2_/g-C_3_N_4_ powder on GCE was used as working, Platinum (Pt) wire as counter and Ag/AgCl (sat. KCl) as reference electrode. All potentials in this work are reported versus Reversible Hydrogen electrode (RHE) according to the Eq. 1.

1 $${\rm{E}}({\rm{RHE}})={\rm{E}}({\rm{Ag}}/{\rm{AgCl}})+0.197+(0.059\times {\rm{pH}})$$

## Results and Discussion

**Figure 1:**
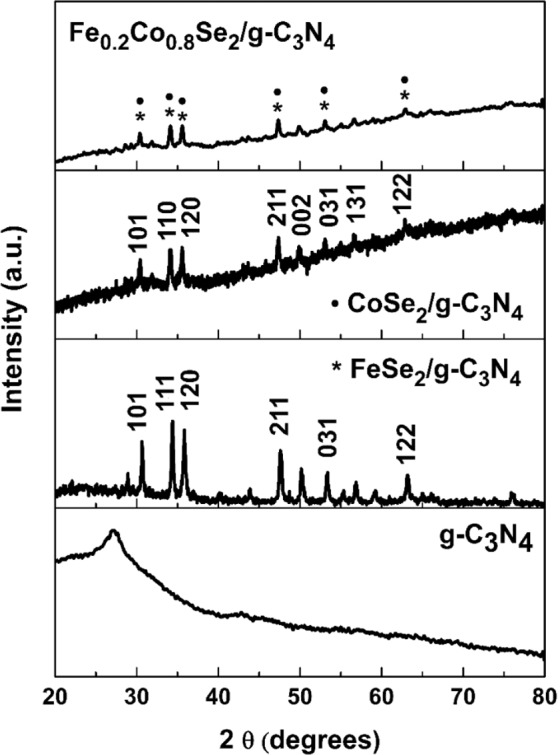
Powder X-ray diffraction pattern of g-C_3_N_4,_ FeSe_2_/g-C_3_N_4_, CoSe_2_/g-C_3_N_4,_ and Fe_0.2_Co_0.8_Se_2_/g-C_3_N_4_.

The Fe/Co ratio was controlled, and a series of samples were prepared. The effect of Fe/Co ratio on the composition and the crystalline phase was investigated by X-ray diffraction (XRD) technique and is shown in Fig. 1. The diffraction peaks at 30.6, 34.4, 35.7, 47.7 and 50.3 match well and can be indexed to the planes of powder diffraction standard database of CoSe_2_ (JCPDS # 53- 0449) and FeSe_2_ (JCPDS # 21- 0432), respectively. It is noteworthy that the diffraction peaks of g-C_3_N_4_ are not indexable in the Fe_0.2_Co_0.8_Se_2_/g-C_3_N_4_ partly due to their comparatively low diffraction intensity, as carbon nitride synthesized from urea shows low diffraction intensity, and partly to relatively small amount of carbon nitride present in the sample. In addition, with increase in Fe amount, the intensity of XRD signals decreased which implied low crystallinity of the Fe_0.2_Co_0.8_Se_2_/g-C_3_N_4_ material in comparison to the pristine ones. Furthermore, the final product (Fe_0.2_Co_0.8_Se_2_/g-C_3_N_4_) showed diffraction peaks corresponding to both CoSe_2,_ and FeSe_2_, which indicates the coexistence of the two orthorhombic phases^52^. It is pertinent to mention here that owing to the small differences in ionic radii of iron and cobalt, the strongest diffraction peaks of FeCoSe_2_, FeSe_2_ and CoSe_2_ displayed very little interlayer spacing difference (±0.01 Å), which provided the basis for facile doping of Fe into the CoSe_2_ structures. Additionally, Fourier transform infrared spectroscopy (FTIR) was conducted to reveal the bonding states of Fe_x_Co_1−x_Se_2_/g-C_3_N_4_ samples (Fig. S1).

**Figure 2:**
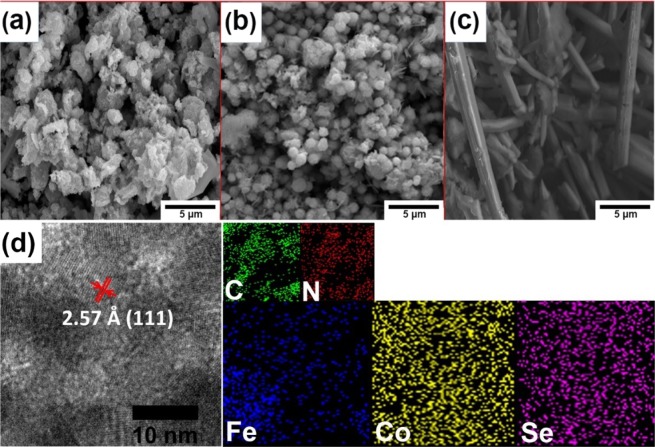
SEM micrographs of (**a**) CoSe_2_/g-C_3_N_4_ (**b**) Fe_0.2_Co_0.8_Se_2_/g-C_3_N_4_ and (**c**) FeSe_2_/g-C_3_N_4_. d) TEM micrograph and elemental mapping images of Fe_0.2_Co_0.8_Se_2_/g-C_3_N_4_.

The morphologies of all samples were studied by SEM (Fig. 2). The Fe free material (CoSe_2_/g-C_3_N_4_) exhibited a not so well-defined nodular morphology (Figs. 2a and S2a), however, after the addition of Fe, Fe_0.2_Co_0.8_Se_2_/g-C_3_N_4_ showed a similar morphology to CoSe_2_/g-C_3_N_4_ but with the appearance of rather uniform sphere like structures embedded on to the CoSe_2_ smooth surface (Fig. 2b). In addition, Fig. 2b depicts a porous framework of Fe_0.2_Co_0.8_Se_2_/g-C_3_N_4_ electrocatalyst, which is favorable for catalytic applications. Whereas, in the case of FeSe_2_/g-C_3_N_4_, due to increase in Fe content in Fe_x_Co_1−x_Se_2_/g-C_3_N_4_, a completely different rod type morphology was seen (Figs. 2c and S3). This appearance of distinct morphologies of two metals (Co, Fe) with Se is as result of their different directional growths under hydrothermal conditions. Furthermore, elemental mapping of synthesized material confirmed a fairly uniform distribution of constituent elements (Fig. S2c and Table S1). The TEM image of Fe_0.2_Co_0.8_Se_2_/g-C_3_N_4_ (Fig. 2d) shows crystalline nature of the material where fringe analysis reveal that the lattice distances remain same throughout the sample. The spherical shape of Fe_0.2_Co_0.8_Se_2_/g-C_3_N_4_, corresponds to the results obtained from SEM.

The as-obtained compounds were further investigated by the simultaneous application of thermogravimetry and differential scanning calorimetry (TG-DSC) to study the effect of temperature, phase transition phenomenon and the associated endothermic or exothermic effects (Figs. S4 and S5). Due to the presence of g-C_3_N_4_ substrate, the compounds are found to be stable for a wide temperature range. Among these samples, g-C_3_N_4_ was found to be the most stable one. While comparing Fe_0.2_Co_0.8_Se_2_ and Fe_0.2_Co_0.8_Se_2_/g-C_3_N_4_, it is clear that g-C_3_N_4_ has a significant effect on the thermal stability of prepared samples. DSC studies of g-C_3_N_4_ reveal that it undergoes an exothermic phase transition in the temperature range of 675–725 °C. Whereas, Fe_0.2_Co_0.8_Se_2_/g-C_3_N_4_ first shows an endothermic transition at 425.6 °C and then an exothermic phase transition at 580 °C. All the samples were further investigated for electrocatalytic overall water splitting performance.

### Electrocatalytic water splitting performance

**Figure 3:**
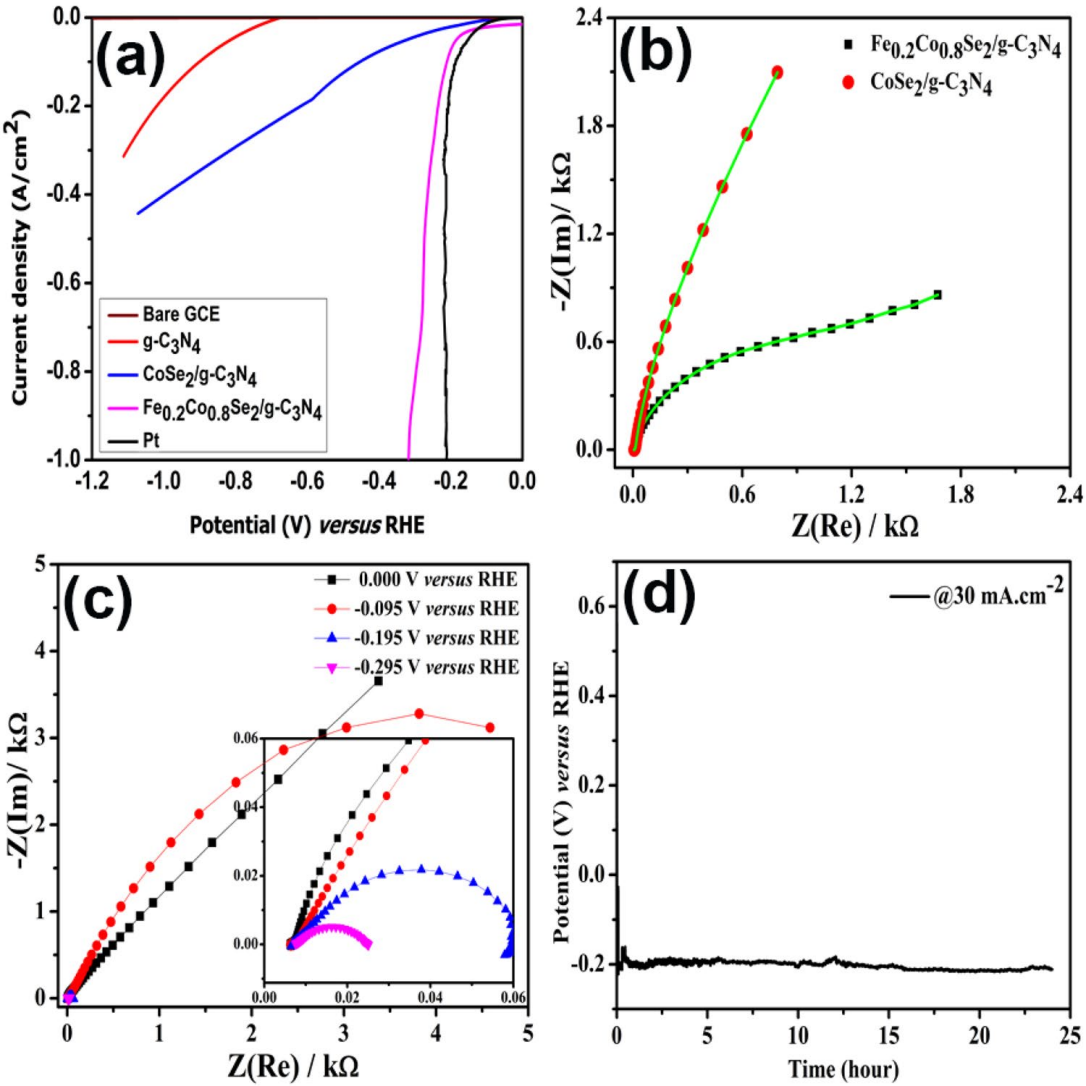
Hydrogen evolution activity of (**a**) bare GCE, g-C_3_N_4_, CoSe_2_/g-C_3_N_4_ and Fe_x_Co_1−x_Se_2_/g-C_3_N_4_ and Pt after iR correction in 0.5 M H_2_SO_4_ (**b**) Nyquist plots for AC impedance measurement of proton conductivity taken under HER working conditions in frequency range of 1000 kHz to 0.1 Hz at 10 mV AC voltage at open-circuit potential (**c**) Nyquist plot of Fe_0.2_Co_0.8_Se_2_/g-C_3_N_4_ at different biases and (**d**) Stability test of Fe_0.2_Co_0.8_Se_2_/g-C_3_N_4_ for 24 h in 0.5 M H_2_SO_4_ (pH 0.3).

The HER performance of the prepared samples Fe_x_Co_1−x_Se_2_/g-C_3_N_4_ (x = 0, 0.2, 0.4, 0.6, 0.8 and 1) were investigated in 0.5 M H_2_SO_4_, DPBS, ASW and 1 M KOH. The best electrocatalytic performance was observed for Fe_0.2_Co_0.8_Se_2_/g-C_3_N_4_. Fig. S6a compares the HER activity of the materials of all composition without iR compensation in 0.5 M H_2_SO_4_. The Fe_0.2_Co_0.8_Se_2_/g-C_3_N_4_ showed best electrocatalytic activity with highest current density values of −14.8, −20, −100 mA cm^−2^ at potential values of 0, 83, and 209 m V respectively in 0.5 M H_2_SO_4_ solution. As HER is facilitated in acidic medium, the current density of −14.8 mA cm^−2^ is likely due to the formation of HSeO_3_^−^ in strong acidic conditions and its subsequent reduction to H_2_Se and or HSe^−^ ^53^. To further explore the role of acid, HER was also carried out in DPBS solution where FeCoSe_2_ exhibited a low current density value of −3.24 mA cm^−2^, confirming the role of acid in the formation and reduction of HSeO_3_^−^ (Fig. S6b)^5^. Basu *et al*. have reported similar results by employing CoSe_2_/C_3_N_4_ catalyst which displayed a photocurrent density of −4.89 mA cm^−2^ ^54^. The Fig. 3a presents the comparison of Fe_0.2_Co_0.8_Se_2_/g-C_3_N_4_ with CoSe_2_/g-C_3_N_4_, g-C_3_N_4_ substrate, and Pt after iR compensation. The poor electrocatalytic HER activity of g-C_3_N_4_ suggests that the major role for higher HER activity is not coming from the substrate itself but it helps in the dispersion of Fe_x_Co_1−x_Se_2_ onto the smooth surface of g-C_3_N_4_.

While CoSe_2_/g-C_3_N_4_ showed comparatively good performance with overpotentials of 193 mV, 450 mV at −20 and −100 mA cm^−2^ respectively than g-C_3_N_4_, the introduction of iron further enhanced the electrocatalytic activity. Therefore, by adjusting the feed ratio, highest and stable HER performance for Fe_0.2_Co_0.8_Se_2_/g-C_3_N_4_ with a current density as high as −1000 mA cm^−2^ was achieved without significant interference from the produced H_2_ bubbles. The negligible interference from the H_2_ bubbles ensures that the modified electrode favors the fast H_2_ desorption from the electrode surface. The fast desorption soon after production is crucial for industrial applications of electrocatalysts (Supplementary video). However, with further increment of the Fe content after optimal ratio (20 wt%), the HER activity sharply decreased due to its probable changes in electronic configurations. In the same way, the electrocatalytic ability of the Fe_0.2_Co_0.8_Se_2_/g-C_3_N_4_ was also assessed in DPBS, ASW, 0.1 M H_2_SO_4_ and 1 M KOH aqueous solutions as shown in Fig. S6b. It is important to note that the relatively high value of −30.12 mA cm^−2^ in 1 M KOH is probably due to a reduction contribution of SeO_3_^2−^ species that are generated at the catalyst-electrolyte interface during testing at high pH conditions. The overall performance of Fe_0.2_Co_0.8_Se_2_/g-C_3_N_4_ is comparable to the benchmark HER electrocatalysts (comparison given in Table S2)^10^.

To further investigate the charge transfer behavior at electrode electrolyte interface, the electrochemical impedance spectroscopy (EIS) of CoSe_2_ and Fe_0.2_Co_0.8_Se_2_ embedded in g-C_3_N_4_ was performed. It can be seen from the Nyquist plots in Fig. 3b, that Fe_0.2_Co_0.8_Se_2_/g-C_3_N_4_ had smaller charge transfer resistance (R_CT_) in comparison with CoSe_2_/g-C_3_N_4_, as the arc diameter of FeCoSe_2_ is smaller than CoSe_2_ dispersed on substrate. Thus, the EIS of Fe_0.2_Co_0.8_Se_2_/g-C_3_N_4_ was further performed at different applied potentials. It can be seen from Fig. 3c that the R_CT_ values decrease with increase in applied potential value. Therefore, further increase in applied potential i.e. −0.295 V decreases the R_CT_ and hence enhances the HER activity. Moreover, determining the electrocatalytic active surface area (ECSA) is an important parameter since it shows the intrinsic performance of electrocatalysts. ECSA was calculated by double-layer capacitance (C_dl_) value (Supplementary note 3) at the electrolyte–electrode interface using cyclic voltammetry (CV given in Fig. S7). As shown in S8, CoSe_2_ increased the C_dl_ of g-C_3_N_4_ from 0.20 and 0.52 mF cm^−2^ to 0.85 and 0.56 mF cm^−2^, respectively at different potential values. After the addition of iron, C_dl_ values rose to 2.73 and 1.20 mF cm^−2^ for Fe_0.2_Co_0.8_Se_2_/g-C_3_N_4._ These results indicate that the introduction of Fe favorably modulates the ECSAs of Fe_x_Co_1−x_Se_2_/g-C_3_N_4_ samples (Table S3).

To assess the stability of Fe_0.2_Co_0.8_Se_2_/g-C_3_N_4_, chronopotentiometric response was recorded at constant current density of −30 mA cm^−2^ (Fig. 3d). The results show that the composite delivers almost an unchanged potential of −0.2 V at −30 mA cm^−2^ for 24 hours. After long term chronopotentiometric response, the samples were subjected to SEM, EDX and elemental mapping. The analysis (Figs. S9 and 10 and Table S1), confirms that after 24 h of testing the constituent elements in the sample and morphology of the synthesized material remained intact. Further, this elemental analysis of hybrid materials was used to find minimum Turn over frequency (TOF_min_), (Supplementary note 2) where TOFs of all catalysts reveal that Fe_0.2_Co_0.8_Se_2_/g-C_3_N_4_ shows highest TOF_min_ (Fig. S6c). All of these results suggest that introduction of iron is of great importance in terms of electrochemical performance as it enhances the charge transfer ability of electrocatalysts and natural hydrogen evolution enzymatic catalysts.

**Figure 4:**
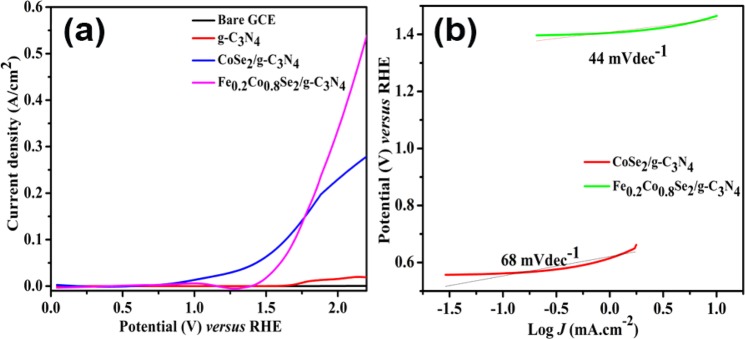
(**a**) OER activity of Bare GCE, g-C_3_N_4_ and Fe_x_Co_1−x_Se_2_/g-C_3_N_4_ in 1 M KOH (**b**) corresponding Tafel slope of CoSe_2_/g-C_3_N_4_ and Fe_0.2_Co_0.8_Se_2_/g-C_3_N_4_ taken at wider potential range in 1 M KOH solution.

The catalytic activity for oxygen evolution reaction (OER) was investigated in aqueous 1 M KOH solution. Fe_0.2_Co_0.8_Se_2_/g-C_3_N_4_ showed moderate overpotentials of 230 and 360 mV at 10 and 50 mA cm^−2^, respectively_._ The results have been compared with recently reported transition metal chalcogenides based electrocatalysts in Table S4 for OER. Moreover, the synthesized material can deliver high current density of over 500 mA cm^−2^ without any interference from the O_2_ bubbles (Fig. 4a). To acquire a direct estimation of intrinsic OER and HER kinetics of electrocatalysts, the Tafel slope was evaluated from the polarization curves. While selecting the region, we avoided very high potentials in order to circumvent oxygen and hydrogen bubble evolution that affects mass transport, and low potentials at which the redox transition for cobalt arises. In each case (i.e., in acidic and alkaline medium), the low Tafel slope of iron incorporated material indicated extremely competitive performance of synthesized material amongst TMDs (Figs. 4b and S11). The Tafel slope values further quantify the enhanced kinetics of charge transfer in the synthesized material.

## Conclusions

A low cost and a facile hydrothermal route was adopted for the preparation of FeCoSe_2_ hybrid uniformly supported on g-C_3_N_4_ that performed efficiently as a bifunctional electrocatalyst. Introduction of iron was found to be of vital importance as it led to increase in TOF_min_ and electrocatalytic ability of nanohybrid material. Due to optimized Fe incorporation, presence of Se atom and a good conducting substrate, the Fe_0.2_Co_0.8_Se_2_/g-C_3_N_4_ ensured high ECSAs and enhanced electrocatalytic performance in the context of low overpotential, small Tafel slope, high current density, high proton conductivity, and stability. In addition, the designed catalyst also performed good OER activity with moderate onset potential and high current density values. Due to facile synthesis technique and good electronic properties, g-C_3_N_4_ can offer a perfect platform for the conversion and modification of catalysts with varied structure and composition. In this work, g-C_3_N_4_ allowed the catalyst to work in wide pH ranges which is highly desirable for industrial applications. These peculiar qualities imply that Fe_0.2_Co_0.8_Se_2_/g-C_3_N_4_ hybrid electrocatalyst has the potential to replace benchmark electrocatalysts for clean fuel production in industrial applications.

## Supplementary information

Supplementary Information.
