# Supplementary material for: FeCoSe2 Nanoparticles Embedded in g-C3N4: A Highly Active and Stable bifunctional electrocatalyst for overall water splitting
Source: Sci Rep. 2020 Apr 14;10:6328. doi: 10.1038/s41598-020-63319-7 (PMC7156446; doi:10.1038/s41598-020-63319-7)
Supplement: Supplementary file 1 — Supplementary Information. [file 41598_2020_63319_MOESM1_ESM.docx]

**FeCoSe_2_ Nanoparticles Embedded in g-C_3_N_4_: A Highly Active and Stable bifunctional electrocatalyst for overall water splitting**

*Muhammad Zulqarnain^1^, Afzal Shah*^,^1,3,* Muhammad Abdullah Khan *^2^, Faiza Jan Iftikhar4 and Jan Nisar^5^*

*^1^* Department of Chemistry Quaid-i-Azam University, 45320, Islamabad, Pakistan

*2*Renewable Energy Advancement laboratory, Department of Environmental Sciences, Quaid-i-Azam University, 45320, Islamabad, Pakistan

^3^Department of Chemistry, College of Science, University of Bahrain, Sakhir 32038, Bahrain

^4^NUTECH School of Applied Sciences and Humanities, National University of Technology, Islamabad 44000, Pakistan.

^5^National Centre of Excellence in Physical Chemistry, University of Peshawar, Peshawar, Pakistan

*To whom correspondence should be addressed

Tel: +92-5190642110

Fax: +92-5190642241

E-mail: [afzals_qau@yahoo.com](mailto:afzals_qau@yahoo.com) (Dr. Afzal Shah)

**Supporting Information**

**Supplementary note 1:**

**Preparation of Dulbecco's Phosphate-Buffered Saline (DPBS)**

Sodium chloride, Disodium phosphate, Potassium chloride and Monopotassium phosphate were mixed in 800 ml of distilled water in the molar ratios of 137, 10, 2.7 and 1.8. Then pH was adjusted at 7.4 with dilute HCl and more water was added to make a final volume of 1 liter^1^.

**Preparation of Artificial Sea Water (ASW)**

Artificial sea water was prepared by following Indian standard artificial sea water preparation methodology^2^. Briefly, Sodium chloride, Magnesium chloride, Sodium sulfate, Calcium chloride, Potassium chloride, Sodium bicarbonate, Potassium bromide, Boric acid, Strontium chloride and Sodium fluoride were dissolved in distilled water with the concentrations of 23.5, 5.0, 3.9, 1.1, 0.66, 0.20, 0.10, 0.026, 0.024 and 0.003 g/L, respectively. Then pH was adjusted to 8.0 by adding few crystals of Sodium carbonate.

**Table S1** EDX spectrum of FeCoSe_2_ before and after stability response.

| **Before stability** | | **After stability** | |
| --- | --- | --- | --- |
| Element | Atomic% | Element | Atomic% |
| O K | 43.49 | O K | 15.91 |
| Fe K | 1.08 | Fe K | 0.46 |
| Co K | 16.26 | Co K | 17.83 |
| Se L | 37.75 | Se L | 62.85 |
| Au M | 1.42 | Au M | 2.95 |

**Table S2.** Comparison of HER activity of Fe_0.2_Co_0.8_Se_2_/g-C_3_N_4_ with other recently reported electrocatalysts in 0.5 M H_2_SO_4_ and 1 M KOH electrolytes.

| **Catalyst** | **Substrate/Electrode** | **Electrolyte** | **Catalyst**  **loading**  **(mg cm^-2^)** | **Current density at 0 V (vs. RHE)** | **Potential**  **mV @**  **10 mA cm^-2^**  **(vs. RHE)** | **Potential**  **mV @**  **20 mA cm^-2^**  **(vs. RHE)** | **Reference** |
| --- | --- | --- | --- | --- | --- | --- | --- |
| **Fe_0.2_Co_0.8_Se_2_** | **g-C_3_N_4_/GCE** | 0.5 M H_2_SO_4_ | 2.12 | -14.80 | ~ | 83 | **This work** |
| **Fe_0.2_Co_0.8_Se_2_** | **g-C_3_N_4_/GCE** | 1 M KOH | 2.12 | -30.12 | ~ | ~ | **This work** |
| **CoSe2** | **g-C_3_N_4_/GCE** | 0.5 M H_2_SO_4_ | 2.12 | 0 | 132 | 193 | **This work** |
| **Hexagonal boron nitride** | **Pt** | 0.5 M H_2_SO_4_ | 0.086 | 0 | 90 | 104 | ^3^ |
| **Ru nanocrystals** | **N-doped C/GCE** | 0.5 M H_2_SO_4_ | 0.28 | 3.1 | 27.5 | 60 | ^4^ |
| **Cobalt phosphosulfide** | **GCE** | 0.5 M H_2_SO_4_ | - | ~2 | 262 | 300 | ^5^ |
| **MoS_2_** | **rGO/GCE** | 0.5 M H_2_SO_4_ | 0.169 | 0 | 96 | - | ^6^ |
| **CoP** | **Co-MOF/GCE** | 0.5 M H_2_SO_4_ | 5 | ~1 | 87 | 135 | ^7^ |
| **Co-Fe-P** | **GCE** | 0.5 M H_2_SO_4_ | 0.285 | 0 | 86 | 109 | ^8^ |
| **Zn-doped MoSe_2_** | **GCE** | 0.5 M H_2_SO_4_ | - | 0 | 231 | 269 | ^9^ |
| **Ni_2_P/MoS_2_/N** | **rGO** | 0.5 M H_2_SO_4_ | 0.526 | ~1.5 | 39.5 | 57 | ^10^ |
| **ReS_2x_Se_2(1-x)_** | **GCE** | 0.5 M H_2_SO_4_ | 0.25 | ~1 | 84 | 100 | ^11^ |
| **Ni-Co-Se** | **NF** | 0.5 M H_2_SO_4_ | 1 | 0 | 150 | ~370 | ^12^ |
| **Co doped WS_2_** | **GCE** | 0.5 M H_2_SO_4_ | 5.1 | 0 | 160 | 183 | ^13^ |
| **CoP** | **N-doped C/GCE** | 0.5 M H_2_SO_4_ | 2 | ~2 | 140 | 158 | ^14^ |
| **Ni_2_P** | **N‐doped C/CC** | 0.5 M H_2_SO_4_ | 0.337 | ~1 | 63.2 | 85 | ^15^ |
| **CoSe_2_** | **NF** | 1 M KOH | 1 | 0 | 84 | - | ^16^ |
| **Tubular CoSe_2_ nanosheets** | **@ NF** | 1 M KOH | 1 | ~2 | 84 | 125 | ^17^ |

**Table S3.** Electrochemical parameters for HER and OER.

| **Catalyst** | **Electrolyte** | **Potential V**  **(vs. RHE)** | **Electrochemical double-layer capacitances**  **(mF)** | **Electrochemical active surface area**  **(cm^2^)** |
| --- | --- | --- | --- | --- |
| **g-C3N4** | 0.5 M H_2_SO_4_ | 0.31 | 0.20 | 5.00 |
| **CoSe_2_/g-C_3_N_4_** | 0.5 M H_2_SO_4_ | 0.31 | 0.85 | 21.25 |
| **Fe_0.2_Co_0.8_Se_2_/g-C_3_N_4_** | 0.5 M H_2_SO_4_ | 0.31 | 2.73 | 68.25 |
|  |  |  |  |  |
| **g-C3N4** | 1 M KOH | 0.50 | 0.52 | 13.00 |
| **CoSe_2_/g-C_3_N_4_** | 1 M KOH | 0.50 | 0.56 | 14.00 |
| **Fe_0.2_Co_0.8_Se_2_/g-C_3_N_4_** | 1 M KOH | 0.50 | 1.20 | 30.00 |
|  |  |  |  |  |
| **Fe_0.2_Co_0.8_Se_2_/g-C_3_N_4_** | 1 M KOH | 0.31 | 0.97 | 24.25 |

**Table S4.** Comparison of OER activity of Fe_0.2_Co_0.8_Se_2_/g-C_3_N_4_ with recently reported transition metal chalcogenides based electrocatalysts in KOH media.

| **Catalyst** | **Substrate/Electrode** | **Electrolyte** | **Catalyst**  **loading**  **(mg cm^-2^)** | **Potential**  **mV @**  **10 mA cm^-2^**  **(vs. RHE)** | **Tafel slope**  **mV dec^-1^** | **Reference** |
| --- | --- | --- | --- | --- | --- | --- |
| **Fe_0.2_Co_0.8_Se_2_** | **g-C_3_N_4_/GCE** | 1 M KOH | 2.12 | 230 | 83 | **This work** |
| **Co_9_S_8_** | **NC/NF** | 1 M KOH | 0.5 | 288 | 65 | ^18^ |
| **PCNs/** | **M_x_S_y_/GCE** | 1 M KOH | 0.51 | 320 | 111 | ^19^ |
| **CoTe_2_@CdTe nanowire array** | **PGE** | 1 M KOH | 2 | 140 | 68 | ^20^ |
| **Co, Fe-codoped NiSe_2_** | **GCE** | 1 M KOH | 0.35 | 268 | 54.78 | ^21^ |
| **MOF Shell and Hollow Ni_3_S_2_/NiS Core** | **GCE** | 0.1 M KOH | 0.2 | 298 | 58.6 | ^22^ |
| **CoSe_2_ nanobelts** | **GCE** | 1 M NaOH | 0.38 | 362.5 | 57.6 | ^23^ |
| **NiCo_2_S_4_/rGO** | **GCE** | 1 M KOH | 0.35 | 366 | 65 | ^24^ |
| **O–CoSe_2_-HNT** | **GCE** | 1 M KOH | 0.35 | 252 | 62 | ^25^ |
| **CoSe_2_/FeSe_2_ DS-HNCs** | **NF** | 0.1 M KOH | 1.8 | 240 | 44 | ^26^ |
| **Co_3_Se_4_&FeSe_2_** | **GCE** | 1 M KOH | 0.14 | 280 | 51 | ^27^ |
| **Co_0.75_Ni_0.25_Se** | **NF** | 1 M KOH | - | 370 | 74 | ^28^ |
| **CoSe_2_ nano-vesicles** | **GCE** | 1 M KOH | 0.25 | 287 | 54.3 | ^29^ |
| **NiSe_2_/g-C_3_N_4_** | **NF** | 1 M KOH | 4 | 290 | 143 | ^30^ |


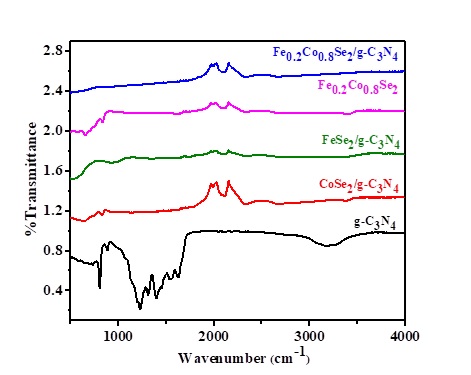


**Figure S1.** FTIR analysis of prepared electrocatalysts.


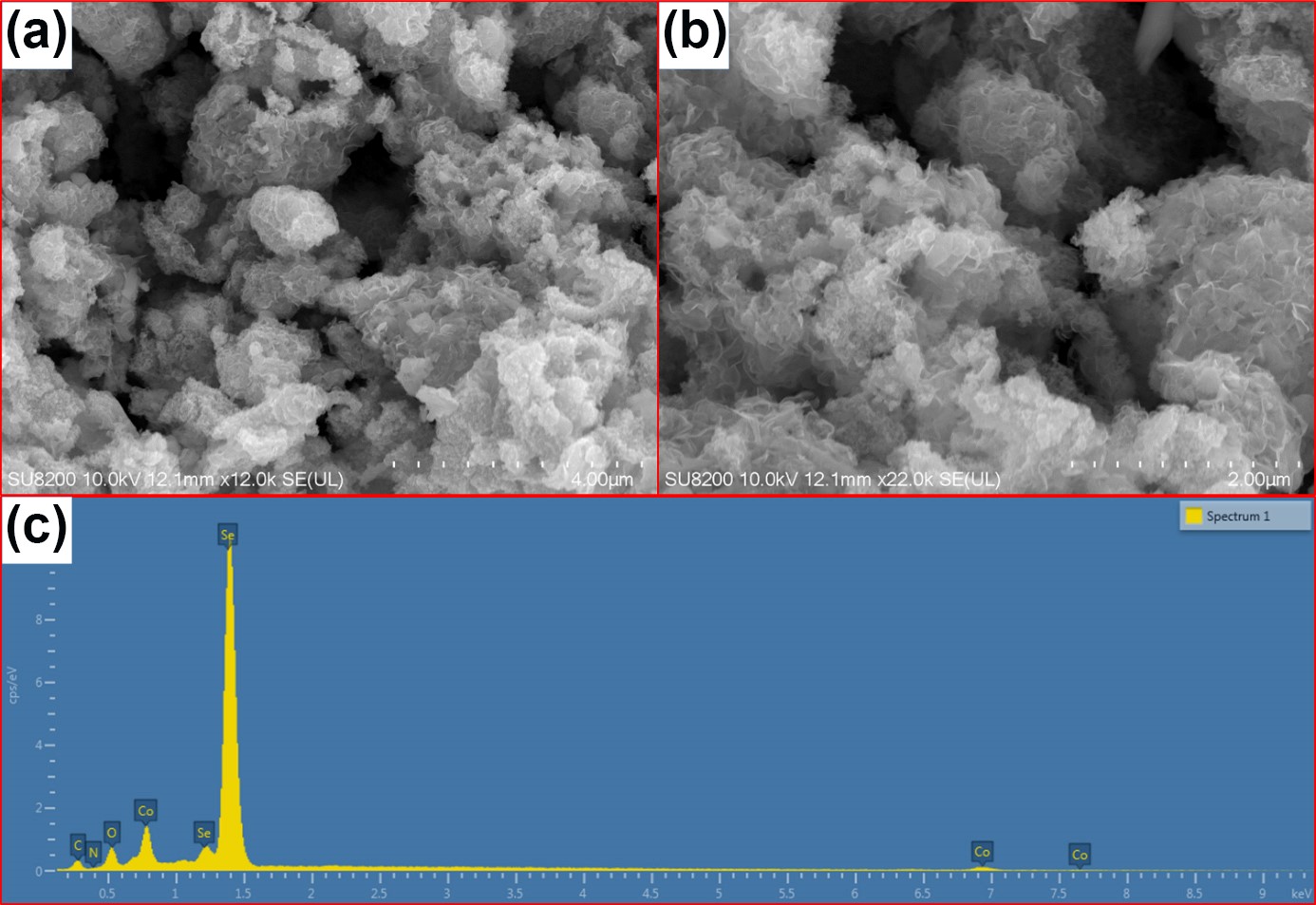


**Figure S2.** SEM of CoSe_2_/g-C_3_N_4_ at a) 2 μm b) 1 μm magnification and c) EDX spectra of CoSe_2_/g-C_3_N_4_.


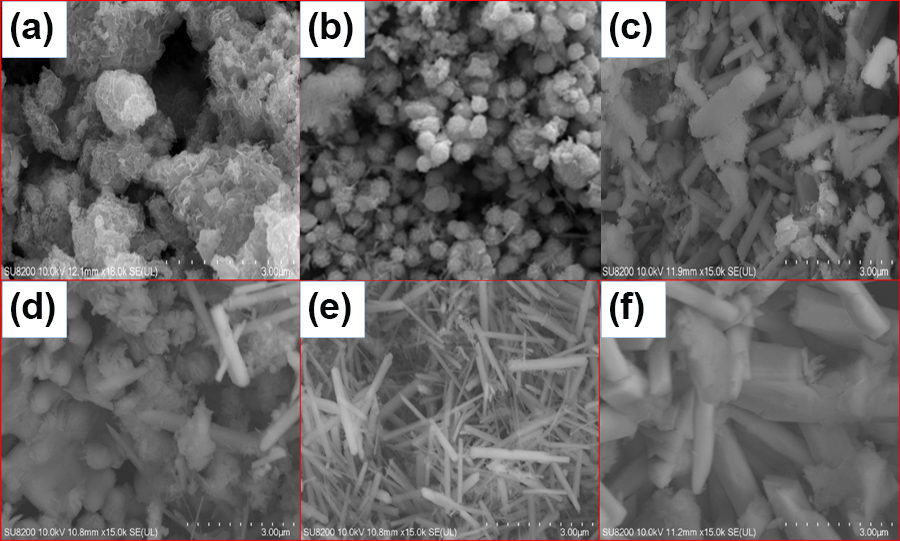


**Figure S3.** SEM micrographs at a) 0 % b) 20 % c) 40 % d) 60 % e) 80 % and f) 100 weight % Fe.


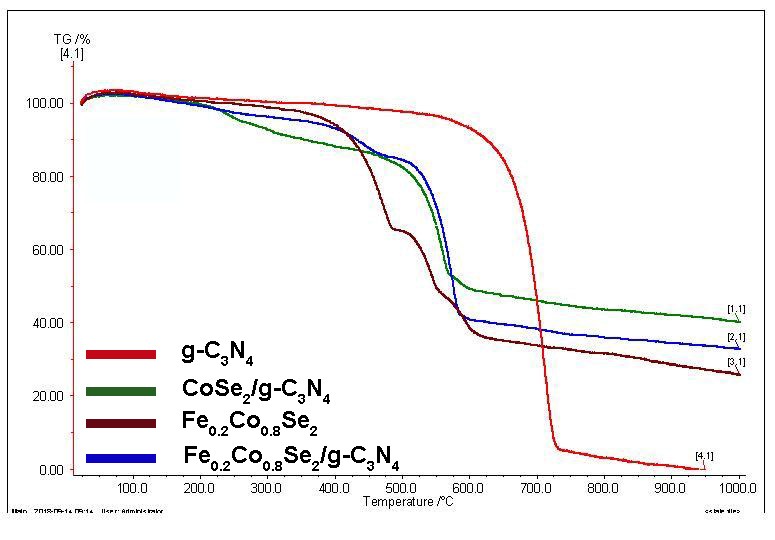


**Figure S4.** Thermogravimetric analysis of g-C_3_N_4_ (red) CoSe_2_/g-C_3_N_4_ (green) Fe_0.2_Co_0.8_Se_2_ (wine) and Fe_0.2_Co_0.8_Se_2_/g-C_3_N_4_ (blue).

**
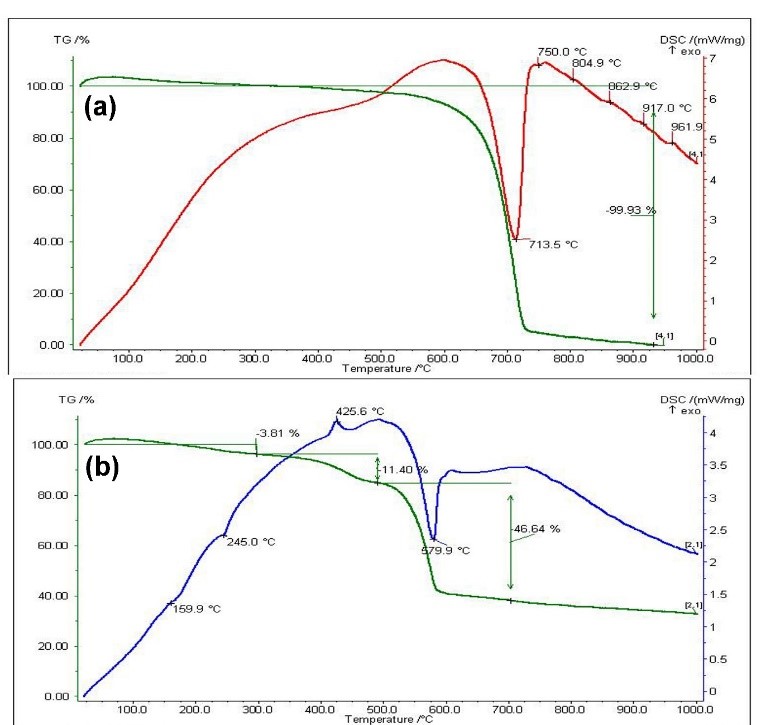
**

**Figure S5.** TGA and DSC studies of a) g-C3N4 and b) Fe_0.2_Co_0.8_Se_2_/g-C_3_N_4_.


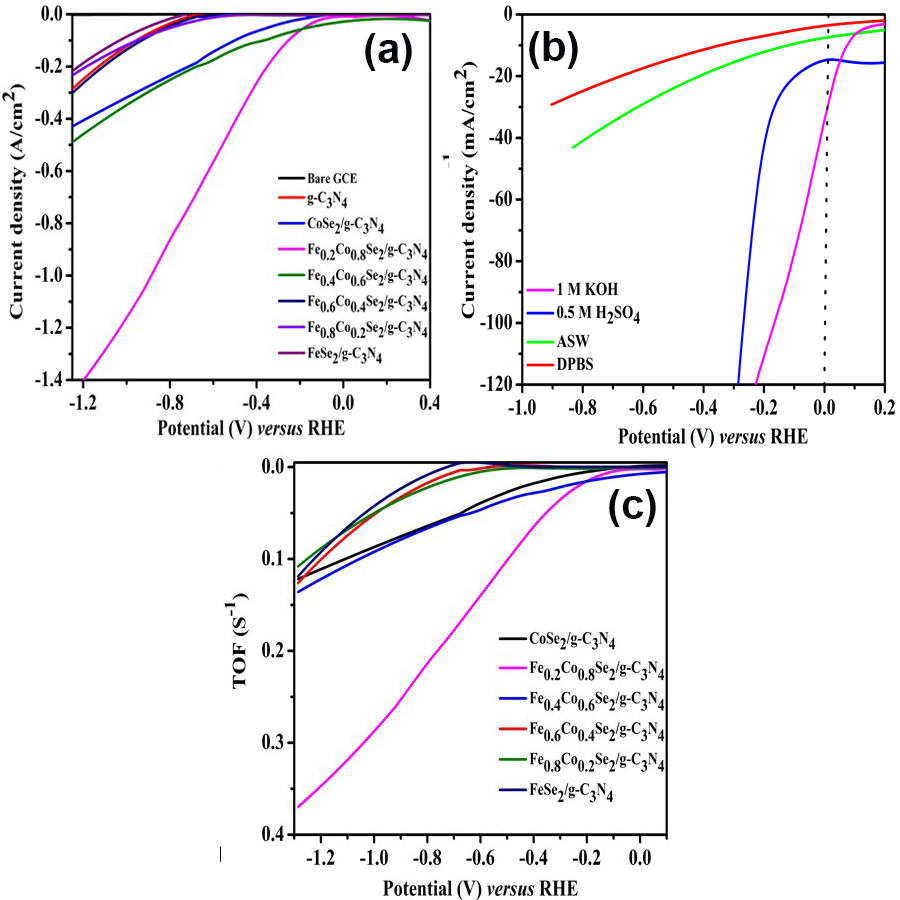


**Figure S6.** (a) HER performance of electrocatalysts at different feeding ratios without iR compensation in 0.5 M H_2_SO_4_ b) HER activity of Fe_0.2_Co_0.8_Se_2_/g-C_3_N_4_ in DPBS (red), Artificial Sea Water (green) 0.5 M H_2_SO_4_ (blue) and 1 M KOH (magenta) after iR compensation and (c) minimum turnover frequency (TOF_min_) of different catalysts at different potentials in HER working condition.

**Supplementary note 2:** Minimum Turn over Frequency (TOF_min_) calculation

Assumptions:

1. Every metal atom present on GCE is accessible for HER
2. It is the lower limit of TOF.

TOF for HER was calculated from the following equation:

$$TOFmin =\frac{Total hydrogen turn over from the geometric surface area of GCE}{Total number of active sites per geometric area of working electrode}$$

$$TOFmin=\frac{J \times A}{4 \times F \times n}$$

Where J is current density (mA cm^-2^), A is geometric surface area (0.0707 cm^-2^), F is Faraday’s constant (96485.3 C mole^-1^) and n is the moles of active sites on GCE calculated by elemental analysis^17^.

**Supplementary note 3:**

Electrocatalytic activity surface area (ECSA) estimation. The ECSA was estimated accoriding to the equation ECSA = C_dl_/C_s_, where C_dl_ denotes electrochemical double-layer capacitance and C_s_ is the specific capacitance of 1 cm^2^ of real surface area. The value of C_s_ is assumed to be 0.04 mF cm^-2^ for the flat electrode. Therefore, cyclic voltammetry was performed in a non-Faradaic region at different scan rates (50, 100, 200, 300 400 and 500 mVsec^-1^). ECSA was estimated directly from the C_dl_ by plotting the Δj = (ja-jc) at different potential as a function of the scan rate.


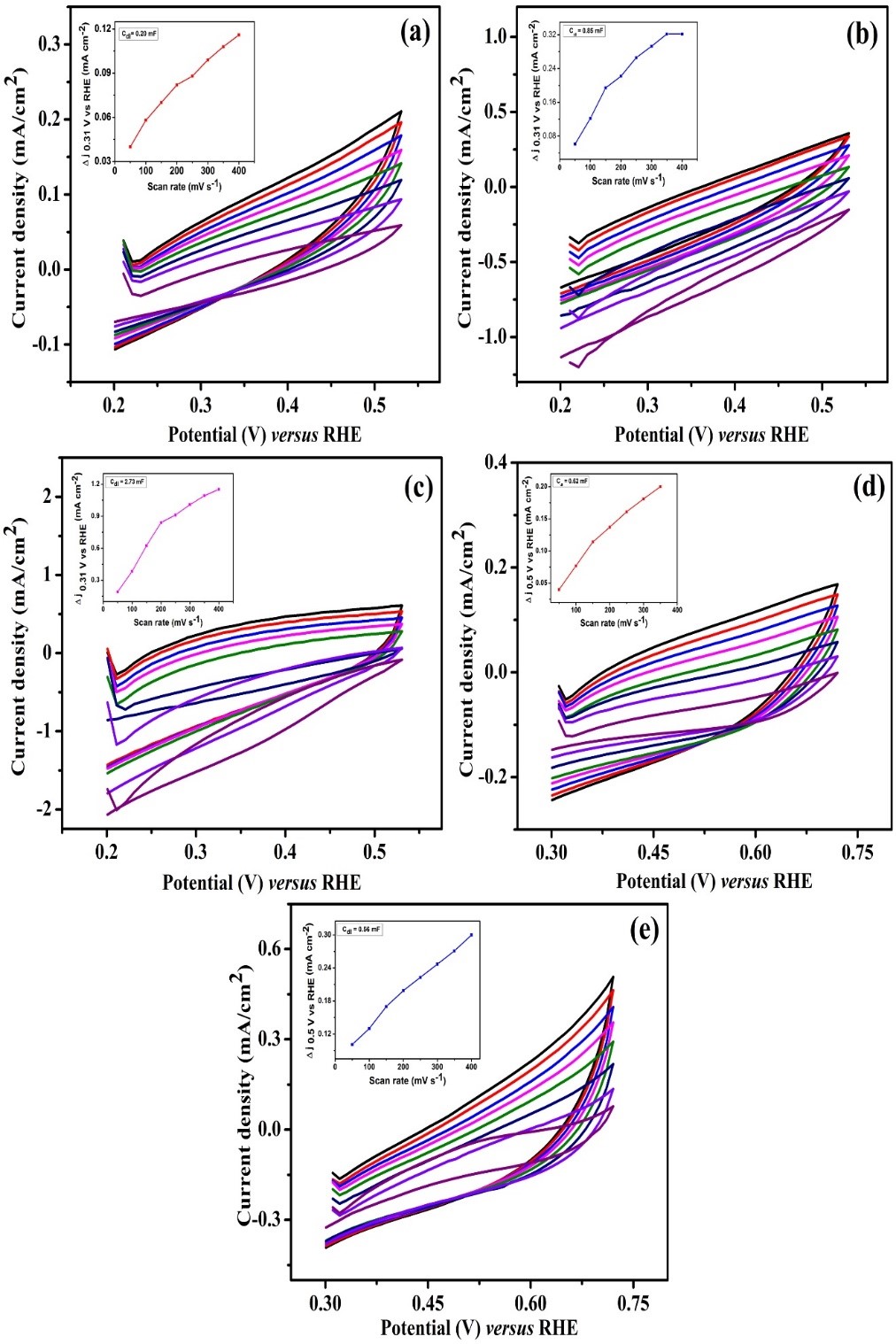


**Figure S7.** Cyclic voltammetry curves of g-C_3_N_4_ (a,d), CoSe_2_/ g-C_3_N_4_ (b,e) and Fe_0.2_Co_0.8_Se_2_/ g-C_3_N_4_ (c,f) in aqueous solutions of 0.5 M H_2_SO_4_ (a,b,c) and 1 M KOH (d and e). Color codes: scan rates of **50** (purple), **100** (violet), **150** (navy), **200** (olive), **250** (magenta), **300** (blue), **350** (red), and **400 mV s^-1^** (black). Inset: images of the scan rate dependence of the current densities for the corresponding materials.


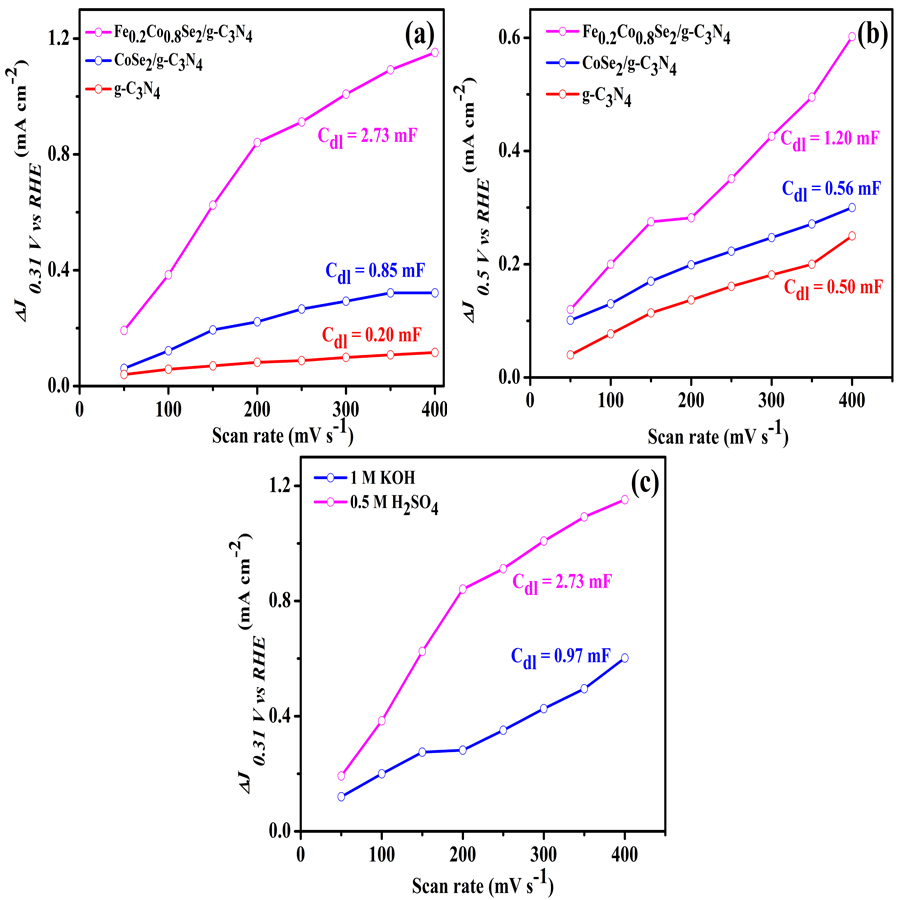


**Figure S8.** Double layer capacitance (C_dl_) calculated from the linear slope between scan rates and ∆j (= j_a_ – j_c_) of different materials in (a) 0.5 M H_2_SO_4_, (b) 0.5 M KOH and (c) Fe_0.2_Co_0.8_Se_2_/g-C_3_N_4_ in both mediums.

**
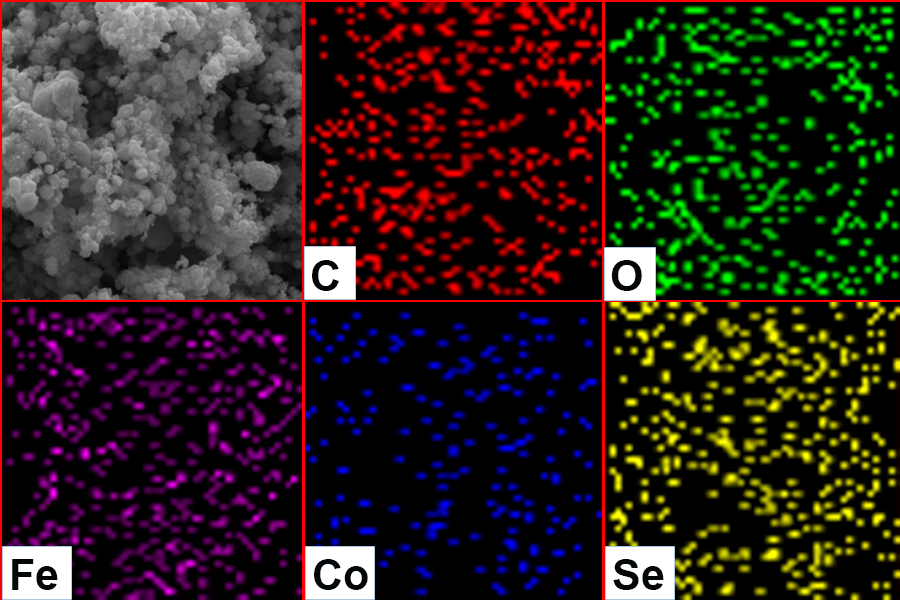
**

**Figure S9.** SEM and elemental mapping of FeCoSe_2_ after chronopotentiometric response for 24 h.


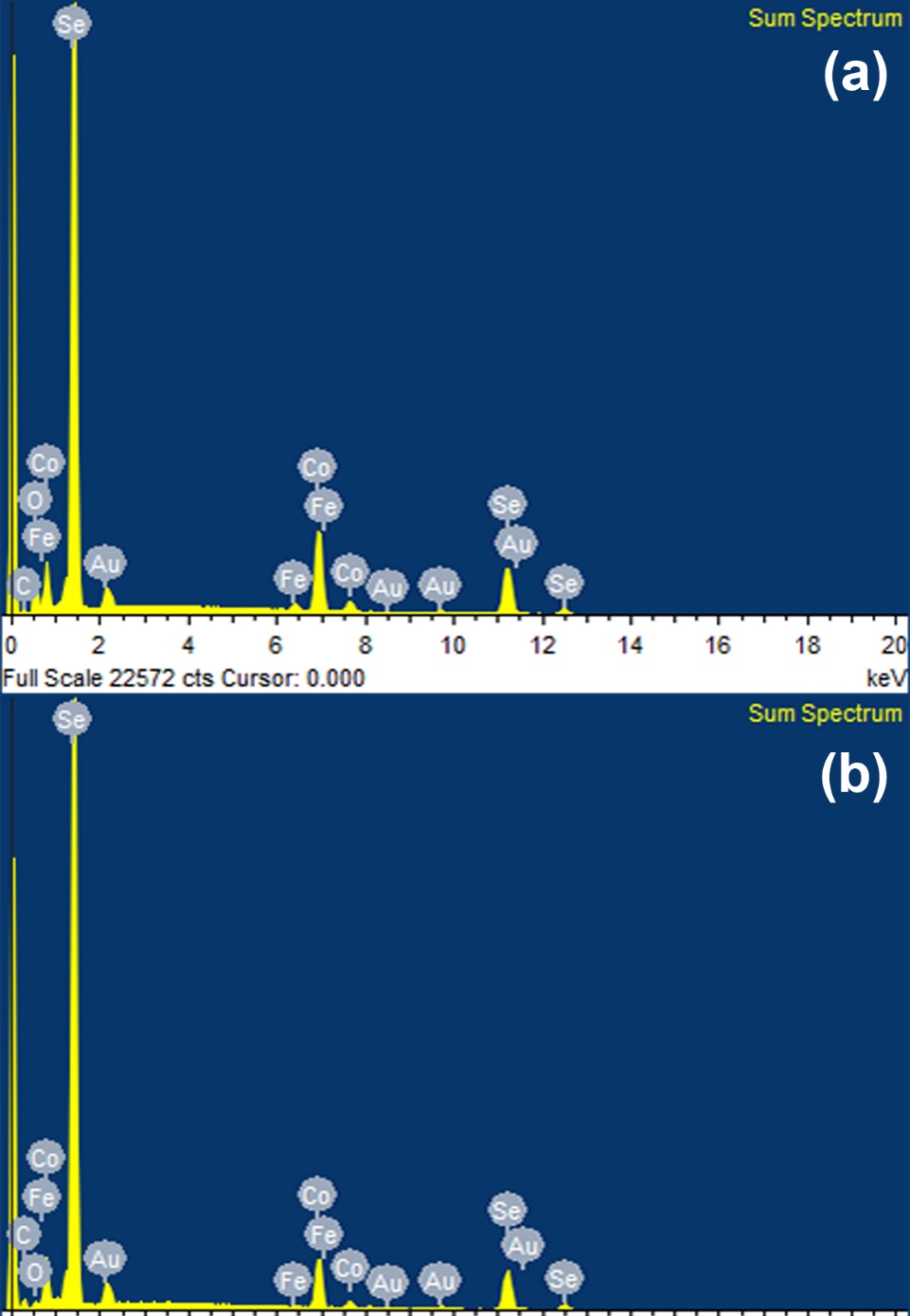


**Figure S10** EDX spectrum of FeCoSe_2_ a) before and b) after 24 h chronopotentiometric response.

**
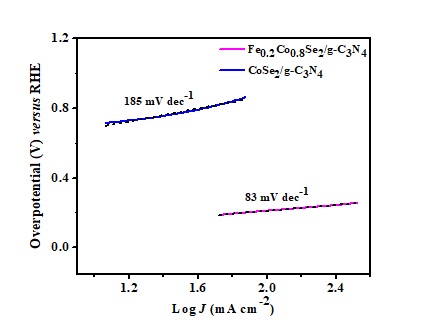
**

**Figure S11.** Tafel slopes for HER taken under wider overpotential range. Color code: CoSe_2_/g-C_3_N_4_ (blue) and Fe_0.2_Co_0.8_Se_2_/g-C_3_N_4_ (magenta).

**References**

1. Dulbecco, R. Plaque formation and isolation of pure lines with poliomyelitis viruses. *J. Exp. Med.* **99**, 167–182 (2004).

2. Kester, D. R., Duedall, I. W., Connors, D. N. & Pytkowicz, R. M. Preparation of artificial seawater. *Limnology and Oceanography* (1967). doi:10.4319/lo.1967.12.1.0176.

3. Guha, A. *et al.* Mechanistic insight into enhanced hydrogen evolution reaction activity of ultrathin hexagonal boron nitride-modified Pt electrodes. *ACS Catal.* (2018). doi:10.1021/acscatal.8b00938.

4. Li, Y. *et al.* Crystallinity dependence of ruthenium nanocatalyst toward hydrogen evolution reaction. *ACS Catal.* **8**, 5714–5720 (2018).

5. Hamers, R. J. *et al.* Crystallographic facet dependence of the hydrogen evolution reaction on cops: Theory and experiments. *ACS Catal.* **8**, 1143–1152 (2017).

6. Tagmatarchis, N., Kagkoura, A., Tasis, D., Dracopoulos, V. & Tzanidis, I. Template synthesis of defect-rich MoS_2_ -based assemblies as electrocatalytic platforms for hydrogen evolution reaction . *Chem. Commun.* (2019). doi:10.1039/c9cc00051h.

7. Cheng, G. *et al.* CoP-doped MOF-based electrocatalyst for pH-universal hydrogen evolution reaction. *Angew. Chemie Int. Ed.* (2019). doi:10.1002/anie.201901409.

8. Chen, J. *et al.* Co-Fe-P nanotubes electrocatalysts derived from metal-organic frameworks for efficient hydrogen evolution reaction under wide pH range. *Nano Energy* **56**, 225–233 (2019).

9. Qian, J., Wang, T., Xia, B., Xi, P. & Gao, D. Zn-doped MoSe_2_ nanosheets as high-performance electrocatalysts for hydrogen evolution reaction in acid media. *Electrochim. Acta* **296**, 701–708 (2019).

10. Kim, M., Anjum, M. A. R., Lee, M., Lee, B. J. & Lee, J. S. Activating MoS_2_ basal plane with Ni_2_P nanoparticles for Pt-like hydrogen evolution reaction in acidic media. *Adv. Funct. Mater.* **1809151**, 1–8 (2019).

11. Zong, Y. *et al.* Preparation of 1T′-Phase ReS_2x_Se_2(1- x )_ (x = 0–1) nanodots for highly efficient electrocatalytic hydrogen evolution reaction . *J. Am. Chem. Soc.* **140**, 8563–8568 (2018).

12. Peng, L. *et al.* Dual tuning of Ni–Co–A (A = P, Se, O) nanosheets by anion substitution and holey engineering for efficient hydrogen evolution. *J. Am. Chem. Soc.* **140**, 5241–5247 (2018).

13. Zhou, L., Yan, S., Song, H., Wu, H. & Shi, Y. Multivariate control of effective cobalt doping in tungsten disulfide for highly efficient hydrogen evolution reaction. *Sci. Rep.* **9**, 1357 (2019).

14. Li, Y. *et al.* Core–shell ZIF-8@ZIF-67-derived cop nanoparticle-embedded N-doped carbon nanotube hollow polyhedron for efficient overall water splitting. *J. Am. Chem. Soc.* **140**, 2610–2618 (2018).

15. Wang, M. Q., Ye, C., Liu, H., Xu, M. & Bao, S. J. Nanosized metal phosphides embedded in nitrogen-doped porous carbon nanofibers for enhanced hydrogen evolution at all pH values. *Angew. Chemie - Int. Ed.* **57**, 1963–1967 (2018).

16. Zhang, J. Y. *et al.* Anodic hydrazine oxidation assists energy-efficient hydrogen evolution over a bifunctional cobalt perselenide nanosheet electrode. *Angew. Chemie - Int. Ed.* **57**, 7649–7653 (2018).

17. Louie, M. W. & Bell, A. T. An investigation of thin-film Ni-Fe oxide catalysts for the electrochemical evolution of oxygen. *J. Am. Chem. Soc.* **135**, 12329–12337 (2013).

18. Sivanantham, A., Hyun, S., Son, M. & Shanmugam, S. Nanostructured core-shell cobalt chalcogenides for efficient water oxidation in alkaline electrolyte. *Electrochim. Acta* (2019). doi:10.1016/j.electacta.2019.04.164.

19. Du, Y. *et al.* In-situ preparation of porous carbon nanosheets loaded with metal chalcogenides for a superior oxygen evolution reaction. *Carbon N. Y.* (2019). doi:10.1016/j.carbon.2019.04.048.

20. Chandra Majhi, K., Karfa, P. & Madhuri, R. Bimetallic transition metal chalcogenide nanowire array: An effective catalyst for overall water splitting. *Electrochim. Acta* (2019). doi:10.1016/j.electacta.2019.06.106.

21. Zhang, J. *et al.* Facile synthesis of NiSe_2_ particles with highly efficient electrocatalytic oxygen evolution reaction. *Mater. Lett.* **235**, 53–56 (2019).

22. Wang, J. & Zeng, H. C. A hybrid electrocatalyst with a coordinatively unsaturated metal-organic framework shell and hollow Ni_3_S_2_/NiS core for oxygen evolution reaction applications. *ACS Appl. Mater. Interfaces* (2019). doi:10.1021/acsami.9b04479.

23. Gui, Y. *et al.* Manipulating the assembled structure of atomically thin CoSe_2_ nanomaterials for enhanced water oxidation catalysis. *Nano Energy* (2019). doi:10.1016/j.nanoen.2018.12.063.

24. Shuai, C. *et al.* Hierarchical NiCo_2_S_4_ nanosheets grown on graphene to catalyze the oxygen evolution reaction. *J. Mater. Sci.* **55**, 1627–1636 (2020).

25. Jia, B. *et al.* Hierarchical nanotubes constructed from CoSe_2_ nanorods with an oxygen-rich surface for an efficient oxygen evolution reaction. *J. Mater. Chem. A* (2019). doi:10.1039/c9ta03606g.

26. Xu, C. *et al.* A facile sequential ion exchange strategy to synthesize CoSe_2_/FeSe_2_ double-shelled hollow nanocuboids for the highly active and stable oxygen evolution reaction. *Nanoscale* (2019). doi:10.1039/c9nr02599e.

27. Wan, J. *et al.* Synthesis from a layered double hydroxide precursor for a highly efficient oxygen evolution reaction. *Inorg. Chem. Front.* (2019). doi:10.1039/c9qi00190e.

28. Liu, S. *et al.* Highly conductive and metallic cobalt-nickel selenide nanorods supported on Ni foam as an efficient electrocatalyst for alkaline water splitting. *Nanoscale* (2019). doi:10.1039/c8nr10545f.

29. Li, Z. *et al.* Facile preparation of CoSe_2_ nano-vesicle derived from ZIF-67 and their application for efficient water oxidation. *Appl. Surf. Sci.* (2019). doi:10.1016/j.apsusc.2019.144368.

30. Wang, S. *et al.* Nanocoral-like composite of nickel selenide nanoparticles anchored on two-dimensional multi-layered graphitic carbon nitride: A highly efficient electrocatalyst for oxygen evolution reaction. *Appl. Catal. B Environ.* (2019). doi:10.1016/j.apcatb.2018.10.071.
